# Draft Genome Sequence of the Deep-Sea Ascomycetous Filamentous Fungus *Cadophora malorum* Mo12 from the Mid-Atlantic Ridge Reveals Its Biotechnological Potential

**DOI:** 10.1128/genomeA.00467-16

**Published:** 2016-07-07

**Authors:** Vanessa Rédou, Abhishek Kumar, Matthieu Hainaut, Bernard Henrissat, Eric Record, Georges Barbier, Gaëtan Burgaud

**Affiliations:** a Université de Brest, Laboratoire Universitaire de Biodiversité et Ecologie Microbienne, Technopôle de Brest Iroise, Plouzané, France; b Division of Molecular Genetic Epidemiology, German Cancer Research Center (DKFZ), Heidelberg, Germany; c Architecture et Fonction des Macromolécules Biologiques, Centre national de la recherche scientifique, Aix-Marseille Université, Marseille, France; d Institut national de recherche agronomique, Marseille, France; e Department of Biological Sciences, King Abdulaziz University, Jeddah, Saudi Arabia; f Institut national de recherche agronomique, Biodiversité et Biotechnologie Fongiques, Aix-Marseille Université, Polytech Marseille, Marseille, France; g Aix-Marseille Université, Biodiversité et Biotechnologie Fongiques, Polytech Marseille, Marseille, France

## Abstract

*Cadophora malorum* Mo12 was isolated from the Rainbow hydrothermal site on the Mid-Atlantic Ridge. We present the draft genome sequence of this filamentous fungal strain, which has high biotechnological potentials as revealed by the presence of genes encoding biotechnologically important enzymes and genes involved in the synthesis of secondary metabolites.

## GENOME ANNOUNCEMENT

*Cadophora malorum* is a filamentous fungus that has been largely reported in Antarctica in lakes (1) or on mosses (2) and even macroalgae (3). Ecophysiological analysis demonstrated that *Cadophora* spp. isolated from Antarctic environments were psychrotrophs (4), popularizing the idea that *Cadophora* sp. strains (including *C. malorum*) were strongly adapted to extreme environments. The strain *Cadophora malorum* Mo12 (UBOCC 108058) was isolated from the Mid-Atlantic Ridge endemic shrimp *Rimicaris exoculata* at the Rainbow hydrothermal site and characterized as a halophilic psychrotrophic fungus (5).

Here, we describe the draft genome sequence of *C. malorum* Mo12 (UBOCC 108058) as a putative producer of original bioactive compounds. High-quality genomic DNA was extracted using the hexadecyltrimethylammonium bromide (CTAB) and Genomic-tip (Qiagen) methods. Genomic DNA of *Cadopora malorum* Mo12 was used to generate shotgun and mate-pair libraries with insert sizes of approximately 350 bp and 8 kb, respectively. A shotgun library was made using the TruSeq DNA PCR-free sample preparation kit and a gel-plus mate-paired-end library was generated with the Nextera mate-pair sample preparation kit. Genome sequencing was performed using Illumina HiSeq 2500 sequencing technologies.

The shotgun library produced 39,507,284 reads. The mate-pair library produced 15,277,956 reads. After quality filtering, reads with more than 90% of bases with base quality greater than or equal to Q20, a total of 28,200,910 shotgun reads (2,848 Mb) and 8,857,004 mate-pair reads (815 Mb), were retained. The ALLPATHS-LG whole-genome shotgun assembler (6) was used for the creation of the *de novo* genome assembly from these short reads. The assembly contained a total of 164 scaffolds with an average read length of 299,210 bp. The *N*_50_ was 1,408 kb, and the maximum contig length was 1,707 kb. The total sequence length of the resulting draft genome was 54,281,849 bp, with an overall GC content of 47.08%. A total of 374,040 bp were repeats, representing 0.76% of the assembled genome size as predicted by repeat masker tool (http://www.repeatmasker.org/). Gene prediction was performed using Augustus 3.0 (7), producing 17,781 protein-coding genes. Our Blast2GO-based annotation analyses (8) have revealed 79% of annotated genes while 21% remained unannotated. The genome analysis of secondary metabolite biosynthesis gene clusters using antiSMASH 3.0 software (9) highlighted the presence of 6 type I polyketide synthases, 5 nonribosomal peptide synthetases, 2 hybrid polyketide synthase/nonribosomal peptide synthesis (PKS/NRPS), and 2 terpene synthases genes. Analysis of the sequence with the CAZy database (10) identified 230 genes with activity involving carbohydrates, including 101 glycoside hydrolases, 74 glycosyltransferases, 1 polysaccharide lyase, 10 carbohydrate esterases, 29 carbohydrate-binding modules, and 15 auxiliary activities. As expected, the array of genes encoding enzymes for the deconstruction of terrestrial plant cell walls is very reduced and the genome does not encode any cellulase. Interestingly, the genome of *Cadophora malorum* Mo12 encodes a candidate alginate lyase, perhaps reflecting adaptation to marine carbohydrates. Due to the significant potential for synthesis of secondary metabolites and the presence of a particular portfolio of genes encoding carbohydrate-active enzymes, we believe that the genome sequence of *Cadophora malorum* Mo12 (UBOCC 108058) will result in the discovery of useful gene products that may be exploited for biotechnological application.

### Nucleotide sequence accession numbers.

The nucleotide sequence of the *Cadophora malorum* UBOCC 108058 (Mo12) genome is deposited in DDBJ/EMBL/GenBank under accession numbers FKJQ01000001 to FKJQ01000591. This paper describes the first version of the genome.

## References

[1] Gonçalves VN, Vaz ABM, Rosa CA, Rosa LH 2012 Diversity and distribution of fungal communities in lakes of Antarctica. FEMS Microbiol Ecol 82:459–471. doi:10.1111/j.1574-6941.2012.01424.x. 22671312

[2] Tosi S, Casado B, Gerdol R, Caretta G 2002 Fungi isolated from Antarctic mosses. Polar Biol 25:262–268.

[3] Furbino LE, Godinho VM, Santiago IF, Pellizari FM, Alves TMA, Zani CL, Junior PAS, Romanha AJ, Carvalho AGO, Gil LHVG, Rosa CA, Minnis AM, Rosa LH 2014 Diversity patterns, ecology and biological activities of fungal communities associated with the endemic macroalgae across the Antarctic Peninsula. Microb Ecol 67:775–787. doi:10.1007/s00248-014-0374-9. 24509705

[4] Duncan SM 2007 Fungal diversity and cellulolytic activity in the historic huts, Ross island, Antarctica. PhD Thesis University of Waikato, Hamilton, New Zealand.

[5] Burgaud G, Le Calvez T, Arzur D, Vandenkoornhuyse P, Barbier G 2009 Diversity of culturable marine filamentous fungi from deep-sea hydrothermal vents. Environ Microbiol 11:1588–1600. doi:10.1111/j.1462-2920.2009.01886.x. 19239486

[6] Gnerre S, MacCallum I, Przybylski D, Ribeiro FJ, Burton JN, Walker BJ, Sharpe T, Hall G, Shea TP, Sykes S, Berlin AM, Aird D, Costello M, Daza R, Williams L, Nicol R, Gnirke A, Nusbaum C, Lander ES, Jaffe DB 2011 High-quality draft assemblies of mammalian genomes from massively parallel sequence data. Proc Natl Acad Sci USA 108:1513–1518. doi:10.1073/pnas.1017351108. 21187386 PMC3029755

[7] Stanke M, Schöffmann O, Morgenstern B, Waack S 2006 Gene prediction in eukaryotes with a generalized hidden Markov model that uses hints from external sources. BMC Bioinformatics 7:62. doi:10.1186/1471-2105-7-62. 16469098 PMC1409804

[8] Götz S, García-Gómez JM, Terol J, Williams TD, Nagaraj SH, Nueda MJ, Robles M, Talón M, Dopazo J, Conesa A 2008 High-throughput functional annotation and data mining with the Blast2GO suite. Nucleic Acids Res 36:3420–3435. doi:10.1093/nar/gkn176. 18445632 PMC2425479

[9] Weber T, Blin K, Duddela S, Krug D, Kim HU, Bruccoleri R, Lee SY, Fischbach MA, Müller R, Wohlleben W, Breitling R, Takano E, Medema MH 2015 antiSMASH 3.0—a comprehensive resource for the genome mining of biosynthetic gene clusters. Nucleic Acids Res 43:W237–W243. doi:10.1093/nar/gkv437. 25948579 PMC4489286

[10] Lombard V, Ramulu HG, Drula E, Coutinho PM, Henrissat B 2014 The carbohydrate-active enzymes database (CAZy) in 2013. Nucleic Acids Res 42:D490–D495. doi:10.1093/nar/gkt1178. 24270786 PMC3965031

